# The Effects of Milrinone Systemic Administration on Cerebral Perfusion as Measured with Computed Tomography in Aneurysmal Subarachnoid Hemorrhage Patients

**DOI:** 10.1007/s12028-026-02559-3

**Published:** 2026-05-28

**Authors:** Vivien Szabo, Nesrine Kabli, Laura Matesanz, Audrey Vilella, Marine Le Corre, Cyril Dargazanli, Chiara Martinez, Pierre-François Perrigault, Quentin Mesnildrey, Kévin Chalard

**Affiliations:** 1 Department of Critical Care Medicine and Anesthesiology, Gui de Chauliac University Hospital of Montpellier, CHU Montpellier, Montpellier, France; 2 IGF, Univ. Montpellier, CNRS, Inserm, Montpellier, France; 3 Department of Neurosurgery, Gui de Chauliac University Hospital of Montpellier, CHU Montpellier, Montpellier, France; 4 Department of Neuroradiology, Gui de Chauliac University Hospital of Montpellier, CHU Montpellier, Montpellier, France

**Keywords:** Subarachnoid hemorrhage, Perfusion computed tomography, Delayed cerebral ischemia

## Abstract

**Background:**

Aneurysmal subarachnoid hemorrhage (SAH) prognosis is determined by the occurrence of delayed cerebral ischemia (DCI), which is associated with specific dynamics of perfusion computed tomography (CTP) parameters. Systemic administration of milrinone is proposed to reduce DCI, though underlying mechanisms remain putative.

**Objective:**

This study aimed to describe the effects of milrinone intravenous infusion on systemic and cerebral hemodynamics in patients with SAH.

**Methods:**

We conducted a retrospective observational study including patients diagnosed with aneurysmal SAH and monitored with PiCCO device and CTP. Relationships between milrinone dose, cardiac index (CI), and CTP parameters were assessed.

**Results:**

A total of 17 patients were included, 88% presented cerebral vasospasm, 29% evolved with DCI. CI increased with systemic milrinone dose in the entire population (*p* < 0*.*001). Contrarily, cerebral blood flow (CBF) decreased with milrinone in the non-DCI subpopulation (*p* = 0*.*002) and was not correlated to it in the DCI one. However, CBF increased with CI in this DCI subpopulation (*p* = 0*.*008). Spatial heterogeneity of perfusion, quantified by the global (CoV) and regional (rCoV) coefficients of variation of CBF, decreased with milrinone dose in the non-DCI subpopulation (*p* < 0*.*001). While no correlation at the global scale emerged in the DCI subpopulation, rCoV increased with milrinone in DCI regions (*p* < 0*.*01).

**Conclusions:**

Intravenous infusion of milrinone did not seem to improve cerebral perfusion through systemic effects. In non-DCI patients, milrinone seemed to homogenize CBF and prevent evolution towards DCI. We suggest that in patients with DCI, it failed to do so, such that CBF spatial heterogeneity pathologically increased until infarction. We propose that spatiotemporal evolution of CBF heterogeneity is both a biomarker and a pathophysiological feature of DCI that advocates for further theoretical and experimental exploration.

**Supplementary Information:**

The online version contains supplementary material available at https://doi.org/10.1007/s12028-026-02559-3.

## Introduction

Patients suffering from aneurysmal subarachnoid hemorrhage (SAH) see their prognosis determined by the occurrence of delayed cerebral ischemia (DCI) [1–4]. Even though DCI pathophysiology’s complexity so far prevents holistic therapeutic approaches, accumulating data suggest that systemic administration of milrinone via intravenous infusion may reduce the risk of DCI [5–8].

Milrinone is a selective antagonist for phosphodiesterase-III. As such, it increases the intracellular concentration in cyclic-AMP and shows pleiotropic effects [9], including positive inotropism [10, 11], arterial vasodilation [10, 12], but also reduction in vascular wall cells pathological proliferation [13], platelet disaggregation [12, 14, 15], and finally may have some antiinflammatory properties [9]. For now, how much of the accomplished clinical outcome in patients with SAH result from indirect systemic and direct cerebral effects can only be speculated [9].

It is now generally admitted that perfusion computed tomography (CTP) parameters display specific spatiotemporal dynamics in patients with DCI [16, 17], including an increase in spatial heterogeneity [18, 19]. In parallel, systemic hemodynamics can be measured using transpulmonary thermodilution [20]. Cerebral blood flow (CBF) being dependent on systemic hemodynamics, it has been suggested that milrinone, through an increase in cardiac output, could increase CBF, and this way improve outcome [9]. However, clinical data are missing to test this hypothesis. In this study, we combined cerebral blood flow and cardiac output measurement to characterize the effects of milrinone intravenous infusion in patients with SAH and describe CTP parameters’ evolution with milrinone administration.

## Methods

### Study Design

We conducted a retrospective study in the neurological Tintensive care unit of Gui de Chauliac Montpellier University Hospital in patients admitted between November 2023 and January 2025. Included patients were all at least 18 years old, diagnosed with SAH (confirmed by CT and angiography). We restricted our study population to patients with both PiCCOO™ monitoring for cardiac output and at least two CTP exams performed during hospitalization. Demographic and clinical data (age, sex, WFNS and Fisher grades, aneurysm location) were also collected.

### Definitions

Cerebral vasospasm (CVS) was defined as a reduction in arteries diameter of at least one-third compared with baseline caliber, measured on either angioCT (CTA) or digital subtraction angiography (DSA).

Delayed cerebral ischemia (DCI) was defined as a delayed ischemic neurologic deficit (DIND) or a delayed cerebral infarction (DCIn) [4].

DIND was characterized by focal neurological impairment or drop in the Glasgow coma scale of at least two points, unexplained by other cause.

DCIn was identified as new infarction on CT or magnetic resonance imaging (MRI) scans, excluding early brain injuries seen within the first 72 h post-aneurysm rupture, and not attributable to other cause.

### Patients’ Management

#### General Management

All patients received healthcare in accordance with current recommendations [21]. Endovascular or surgical aneurysm securization was decided by neurosurgeons and neuroradiologists. Surgical procedures including external ventricular drainage (EVD), intracerebral hemorrhage evacuation or decompressive craniectomy were performed when necessary. Comatose patients were sedated and assisted with mechanical ventilation. All patients received nimodipine, administered either orally every 4 h in a total daily dose of 360 mg, or intravenously in a dose of 2 mg/h when the oral route was not available. Patients were under clinical supervision and transcranial Doppler (TCD) monitoring to detect neurological deterioration and/or elevated velocities in the cerebral arteries. Systematic baseline CTP were performed during the first 72 h after aneurysm securization [8]. When suspected after clinical examination and/or TCD, CVS and DCIn were evaluated on CT scans. In patients with CVS and/or DCI, cerebral perfusion pressure was optimized by maintaining normovolemia. A CTP-guided systemic administration of milrinone in both preventing and treating DCI was performed. Intra-arterial administration of both nimodipine and milrinone, or percutaneous transluminal angioplasty were used as a last resort.

#### Milrinone Protocol

Intravenous administration (IV) of milrinone was based on CTP evaluation of brain perfusion, according to the following protocol [8]. IV milrinone was initiated when CTP showed an increase in Time-to-Maximum (TMax) and/or Mean Transit Time (MTT) compared with baseline values. Initial dose was 0.5 µg/kg/min. CTP re-evaluation was then performed every 1 or 2 days until TMax and/or MTT stabilization or normalization. When perfusion deterioration persisted, IV milrinone dose was increased by steps of 0.5 µg/kg/min daily, to a maximum of 2 µg/kg/min.

#### Milrinone Data Collection

The administered dose was recorded for each patient throughout the stay and expressed in µg/kg/min. Metabolic integration was considered instantaneous and not cumulative (*i.e.* transient integration time and metabolic accumulation were neglected). As a result, the actual dose at any time point could directly be inferred from the corresponding posology.

#### Cardiac Index and PiCCO

Cardiac index (CI), computed as the ratio of cardiac output over body surface area, was obtained using a PiCCO device and performed transpulmonary thermodilution, using the mean of three measurements [22], four times a day, and was expressed in ml/min/m^2^, as part of routine clinical practice. We used data produced by embedded PiCCO software, that uses a modified Stewart–Hamilton algorithm. Pulse contour analysis provided systemic vascular resistance (iSVR). It has been proposed that CI and CBF may covary after SAH [23], such that an increase in CI (may it be due to milrinone or not) would lead to an increase in CBF and brain oxygenation, and this way reduce DCI occurrence [9, 24].

#### CTP Acquisition and Processing

CTP exams were acquired on a GE24-slice scanner with a 5-mm slice thickness. Cerebral blood flow (CBF) and Time-to-Maximum (TMax) maps were calculated using Perfusion4D software (GE Healthcare, *revo ct* 22*bc.*50). The basilar artery was used as the arterial input function. All images were anonymized and converted to NIFTI (Neuroimaging Informatics Technology Initiative) format using SPM12 (Statistical Parametric Mapping, (https://www.fil.ion.ucl.ac.uk/spm/). NIFTI files were registered into the MNI space (Montreal Neurological Institute, [25] using ANTsPy library [26] for Python software, and the high-resolution scCT template (stroke-control CT, [27]) was used as the target template. For each exam, we computed the following transformation fields using the “SyN” method (ANTsPy library):

- CTP to anatomical CT (Tx1 transformation);
- Anatomical CT to scCT Template (Tx2 transformation);

Transformations Tx1 and Tx2 were then consecutively applied to the original parametric maps. Finally, image registration quality was assessed by the authors.

Postprocessing of CTP data was conducted using a custom analysis pipeline developed in MATLAB (The MathWorks, Natick, CA, USA, v2024b) [19]. For each patient, parametric perfusion maps (including CBF and TMax) were processed and segmented using a standardized arterial atlas [28]. We defined the whole-brain volume as the aggregate of six main vascular territories: the bilateral anterior cerebral arteries (ACA), middle cerebral arteries (MCA), posterior cerebral arteries (PCA). After segmentation, the mean, the standard deviation (STD), and the coefficient of variation (the ratio of STD over mean) were calculated both at the whole-brain and at the arterial region of interest (ROI) scale. The whole-brain coefficient of variation and regional coefficient of variation are referred to as CoV and rCoV, respectively, in the following. The CoV quantifies the variability of a given parameter; it here allows to quantify cerebral perfusion’s spatial heterogeneity, which may be hidden by spatial averaging used for providing mean Tmax and mean MTT. An increase in such perfusion spatial heterogeneity has been shown to precede DCIn [19].

### Multimodal Analysis and Statistics

Relationships between hemodynamical and cerebral perfusion metrics were investigated in several groups. At the whole-brain level, we considered the whole population of patients (Global), patients who were not diagnosed with DCI (non-DCI group), and patients who were diagnosed with DCI (DCI group). At the arterial ROI level, when analyzing rCoV, we distinguished three categories: (1) ROIs from the non-DCI population of patients (no-DCI-ROIs(non-DCI patients)), (2) ROIs where DCIn was objectively assessed (DCI-ROIs), and those which showed no evidence of infarction (noDCI-ROI(DCI patients)) within the DCI population of patients. To account for the fact that perfusion and hemodynamical metrics were not recorded simultaneously and at different sampling rates (every 2 h for hemodynamics), hemodynamic data were first smoothed using a moving average method and a span window of 12 hours. Downsampled data were then interpolated from the corresponding smoothed trends at the same time points as the actual perfusion data. Different sample sizes were thus considered depending on the analysis. All data were time-referenced to the date of symptom onset (day 0), defined by first clinical signs such as severe headache, loss of consciousness, or vomiting. Analyses were performed using custom scripts in MATLAB (The MathWorks, Natick, CA, USA, v2024b). Linear relationships between hemodynamics, milrinone dose, and perfusion metrics were investigated using Pearson’s correlation statistics. In each case, the slope of the linear trend (noted *a*), the *r*^2^ and *p* − value are reported.

## Results

### Patients’ Description

A total of 17 patients with SAH were included in the study. Among them, 15 patients (88%) developed cerebral vasospasm (CVS), with a mean onset at 5.3 ± 2.3 days after the initial hemorrhagic event. Furthermore, five patients (29%) developed DCI, all in the form of DCIn, with a mean onset at 10.3 ± 2.4 days. One patient was under high flow nasal canula, whereas all other patients were under mechanical ventilation. Patients had between 4 and 13 CTP (6.8 on average) performed with a mean delay of 2.3 days (ranging from 1.3 and 3.6 days). Details are provided in Table 1. Median values of hemodynamical data as well as average drug dose per 24 h time slots are reported in Supplementary Table 1 for all subjects.

**Table 1:** Population description

Total patients	17
Female	10 (59%)
Male	7 (41%)
Age, years	53 (27–74)
Modified Fisher score	
1–2	0 (0%)
3–4	17 (100%)
WFNS	
I-II	5 (29%)
III-V	12 (71%)
Aneurysm location	
Anterior communicating artery or anterior cerebral arteries	9 (53%)
Middle cerebral arteries	4 (23%)
Posterior communicating arteries or internal carotids	1 (6%)
Posterior cerebral arteries	1 (6%)
Vertebro-basilar arteries	2 (12%)
Vasospasm occurence	15 (88%)
Vasospasm delay, days	5.3 (0.9–9.6))
Delayed cerebral ischemia occurence	5 (29%)

### Cardiac Index and Cerebral Blood Flow

#### Cardiac Index

**Fig. 1:**
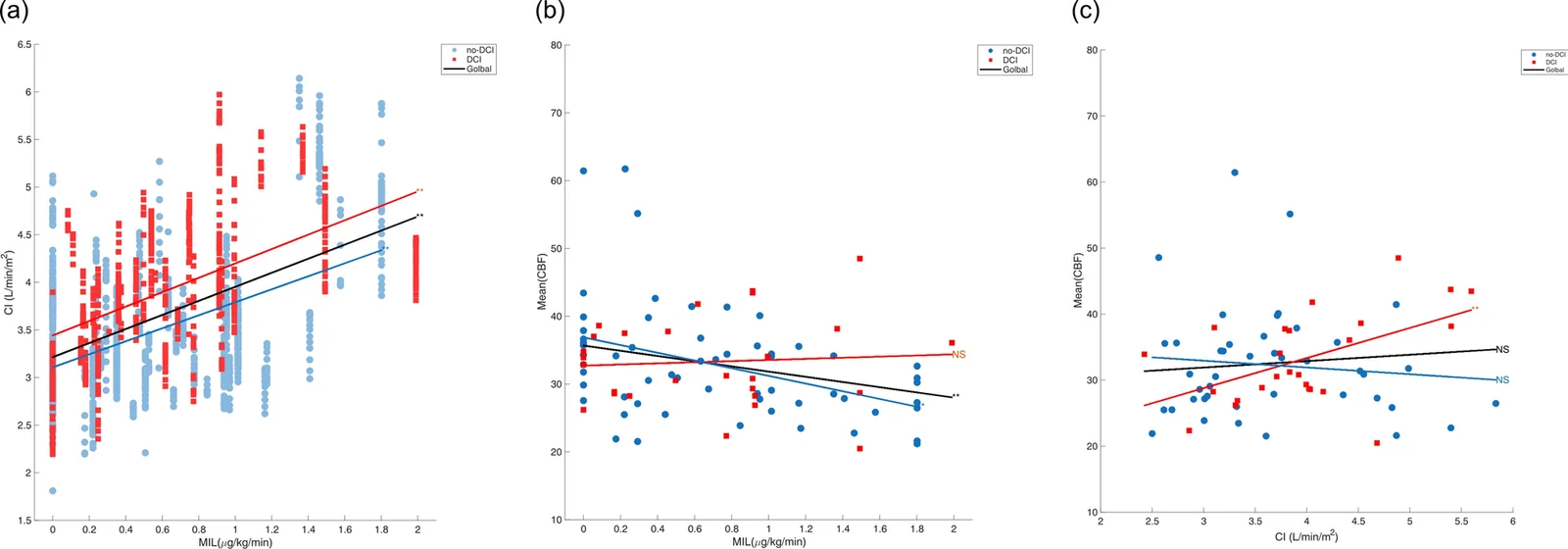
**a** Cardiac index as a function of the dose of milrinone. **b** Mean CBF as a function of the dose of milrinone. **c** Mean CBF as a function of cardiac index. Red squares and blue dots represent data from the DCI and non-DCI subpopulations, respectively. Lines represent the linear correlation between datasets. Colored lines refer to DCI and non-DCI subpopulations, and the black curve refers to the entire population. Stars at the end of the line indicate a statistically significant relationship (Color figure online)

Cardiac index increased with mirlinone dose. This was the case when considering the whole population (*a* = 0*.*70, *R*^2^ = 0*.*22, *p* < 0*.*001 Fig. 1a), as well as in both the DCI and non-DCI subpopulations (*a*_DCI_ = 0*.*70, *R*^2^ = 0*.*26, *p* < 0*.*001 and *a*_noDCI_ = 0*.*65, *R*^2^ = 0*.*21, *p* < 0*.*001, respectively).

#### Cerebral Blood Flow

Mean CBF in the whole population decreased with milrinone dose (Fig. 1b). However, DCI and non-DCI patients showed distinct trends. While CBF decreased in non-DCI patients, no relationship was found in DCI ones (a_noDCI_ = −5.68, R2 = 0.16, p = 0.002).

#### Relationship Between CBF and CI

Similarly, the relationships between mean CBF and CI were different in the two categories of patients (Fig. 1c). They were not correlated in the whole population and in the non-DCI patients. However, mean CBF increased with CI in the DCI subpopulation (*a*_DCI_ = 4*.*55, *R*^2^ = 0*.*27, *p* = 0*.*008).

#### Effect of Baseline iSVR

The positive correlation found between CI and milrinone dose was conserved when categorizing data according to baseline iSVR (Supplementary Fig. S1a). Besides, no significant relationship was found in any baseline iSVR category between mean CBF and milrinone dose (Supplemetary Fig. S1b).

### Cerebral Perfusion Spatial Heterogeneity

#### Whole-Brain Coefficient of Variation of CBF

**Fig. 2:**
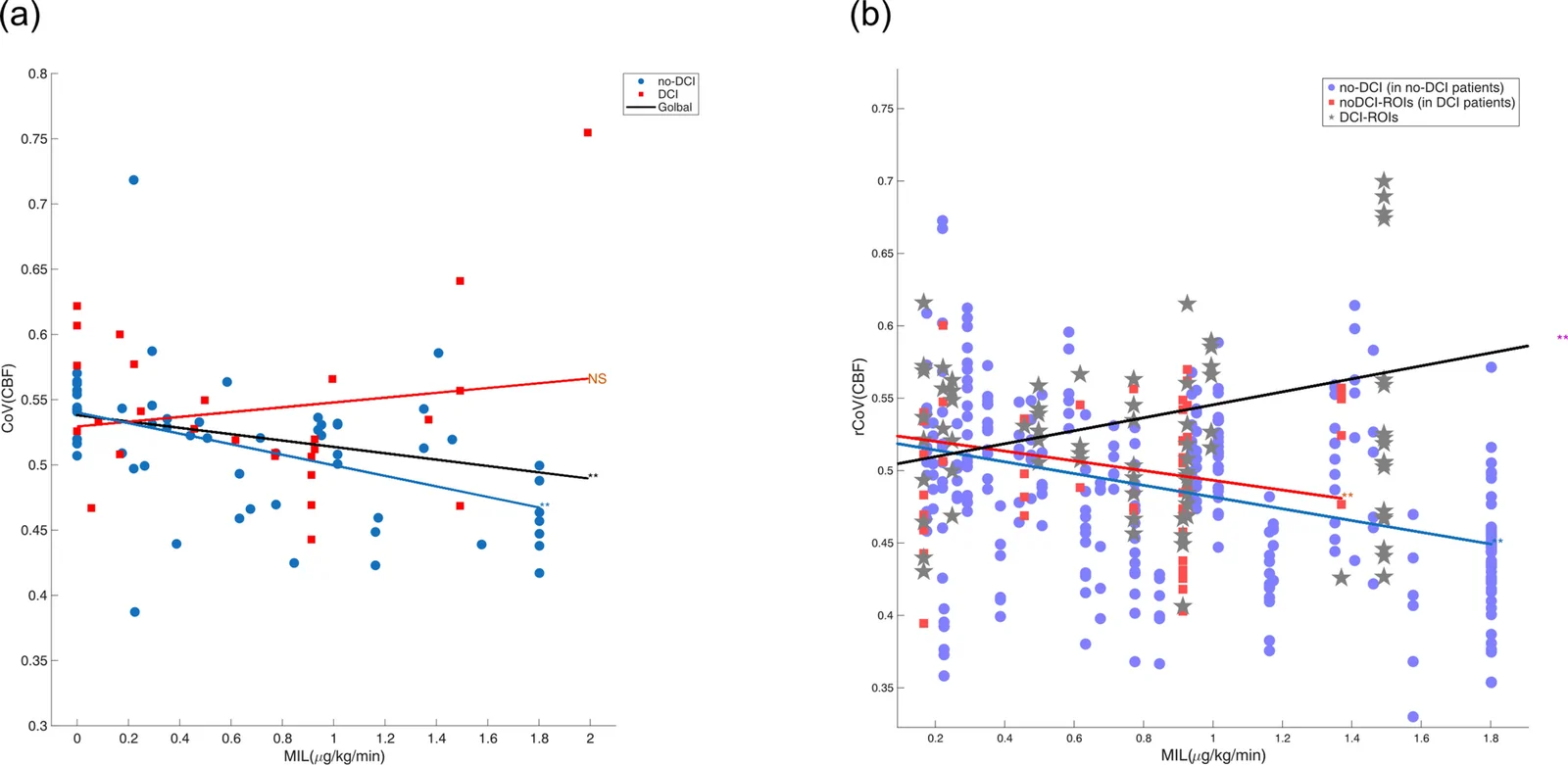
**a** Whole-brain coefficient of variation of CBF, CoV, as a function of the dose of milrinone. Red and blue dots represent data from the DCI and non-DCI subpopulations, respectively. **b** Regional coefficient of variation of CBF, rCoV, as a function of the dose of milrinone for pooled arterial ROIs per category. Blue dots represent data from non-DCI patients, red squares represent no-DCI ROIs from the DCI patients population, and black stars represent DCI-ROIs. Lines represent the linear correlation between datasets for the three subpopulations. Stars at the end of the line indicate a statistically significant relationship (Color figure online)

Spatial coefficient of variation (CoV) of CBF decreased with the dose of milrinone (*a* =  − 0*.*02, *R*^2^ = 0*.*06, *p* = 0*.*02, Fig. 2a). This relationship found in the whole population was due to the correlation in the non-DCI category of patients (*a*_noDCI_ =  − 0*.*04, *R*^2^ = 0*.*21, *p* < 0*.*001); no such correlation was found in the DCI one (*a*_DCI_ = 0*.*018, *R*^2^ = 0*.*03, *p* = 0*.*42).

#### Regional Coefficient of Variation of CBF

At the arterial-ROI level, the relationship between rCoV and the dose of milrinone exhibited different behaviors in the three subcategories of data. In the non-DCI group, rCoV significantly decreased with the dose of milrinone for pooled ROIs (*a*_noDCI_ =  − 0*.*041, *R*^2^ = 0*.*19, *p* < 0*.*001, Fig. 2b). For individual arterial ROIs, a significant negative linear relationship was also found for bilateral ACA, PCA but not for MCA (Supplementary Fig. S2a).

In DCI-ROIs, rCoV significantly increased with the dose of milrinone for pooled. ROIs (*a*_DCI−ROIs_ = 0*.*045, *R*^2^ = 0*.*12, *p* < 0*.*01). Interestingly, no-DCI ROIs from the DCI patient population exhibited a behavior similar to the non-DCI patients data, with a significant decrease of rCoV with the dose of milrinone (*a*_noDCI_ − _ROIs(DCIpatients)_ =  − 0*.*034, *R*^2^ = 0*.*07, *p* < 0*.*01). For individual arterial ROIs, a significant positive linear relationship was found for the right-ACA and left-MCA. A increasing trend was also observed for the left-ACA, right-MCA, and left-PCA but failed to reach a significance level. For the right-PCA, a non-significant negative trend could be observed. (Supplementary Fig. S2a).

#### Regional Coefficient of Variation of TMax

Evolution of spatial distribution of TMax with milrinone dose was similar to that of CBF. Specifically, whole-brain CoV of TMax decreased with milrinone dose in the non-DCI subpopulation (*a*_noDCI_ =  − 0*.*031, *R*^2^ = 0*.*073, *p* = 0*.*040, Supplementary Fig. S3a). rCoV of TMax showed a decrease in no-DCI ROIs from non-DCI patients for pooled ROIs (*a*_noDCI_ =  − 0*.*027, *R*^2^ = 0*.*032, *p* < 0*.*001). Again, for DCI-ROIs, rCoV increased for pooled ROIs (*a*_DCI−ROIs_ = 0*.*045, *R*^2^ = 0*.*050, *p* = 0*.*02, Supplementary Fig. S3b). For individual ROIs, a positive linear relationship was observed between rCoV and milrinone but only reached significance level in MCA territories (Supplementary Fig. S2b).

#### Temporal Evolution

**Fig. 3:**
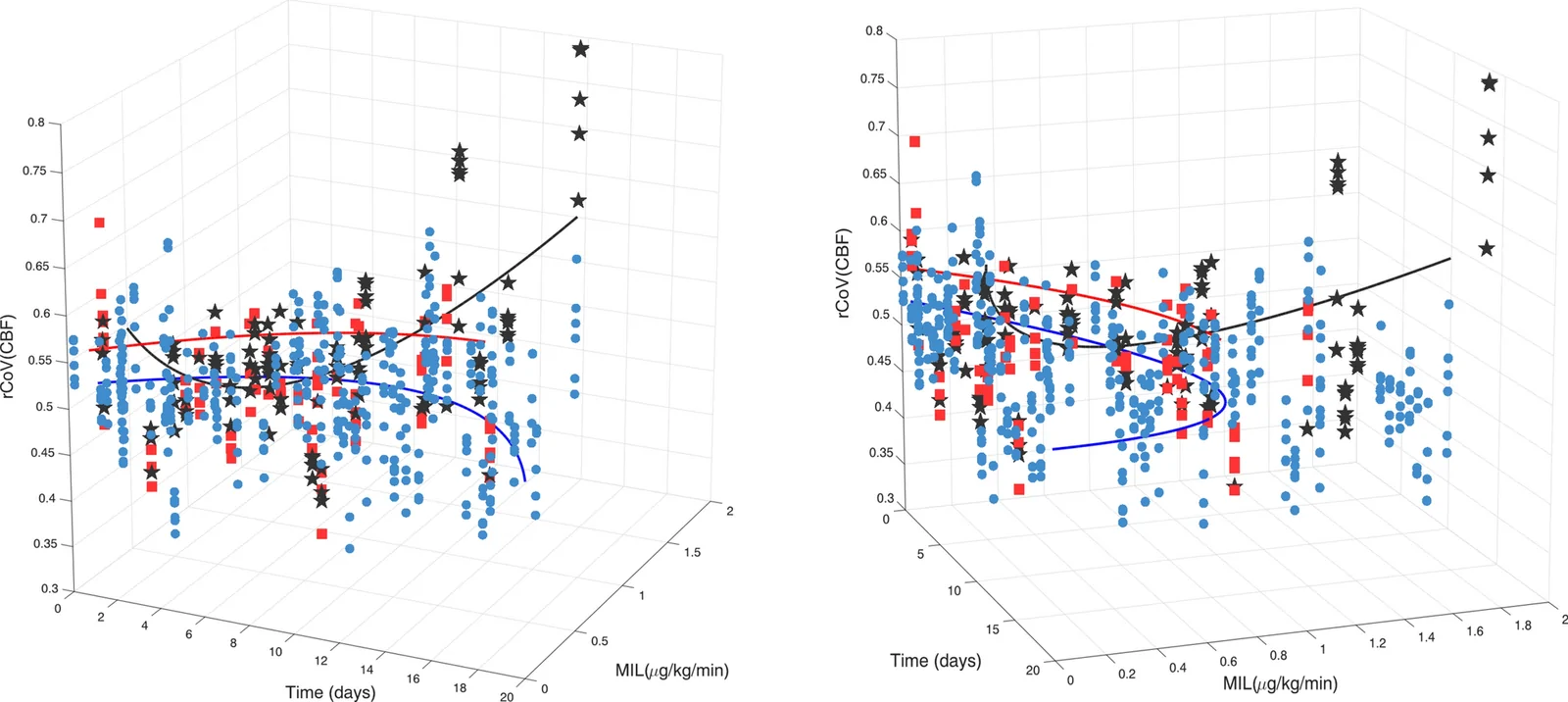
Two different views of 3D-representation of rCoV as a function of the milrinone dose and time for DCI-ROIs (black stars), no-DCI-ROIs from non-DCI patients (blue dots), and no-DCI ROIs from DCI patients (red squares). Solid lines result from a second-degree polynomial fit of the rCoV-Milrinone relationship as a function of time for all three categories of data (Color figure online)

Figure 3 illustrates the relationship between rCoV and milrinone dose as a function of time in three categories: no-DCI ROIs from non-DCI patients (blue dots and fitted curve), no-DCI ROIs from DCI patients (red squares and fitted curve), and DCI-ROIs (black stars and fitted curve).

In non-DCI patients, the dose of milrinone gradually increases with time, reaching its peak around day 10 following SAH, while rCoV decreases. After day 10, rCoV remains stable and the dose of milrinone decreases with time.

In DCI patients, initial rCoV values are larger than in the no-DCI population. The dose of milrinone increases in the following days, while rCoV shows a moderate decrease. After day 10, different patterns emerge from the no-DCI and DCI-ROIs. In no-DCI-ROIs from DCI patients, rCoV stabilized at higher values than in the nonDCI patients. In contrast, rCoV monotonously increased in DCI-ROIs with milrinone dose and time, and finally infarcted.

## Discussion

Our data allow, first, to characterize systemic and cerebral hemodynamics in SAH patients in response to intravenous infusion of milrinone [8] and second, to describe cerebral perfusion’s dynamics associated with DCI. In this cohort, otherwise similar to previously reported ones [1, 8], DCI may have been higher than in a previous report [8]; this can be explained by a selection bias. Indeed, PiCCO monitoring was performed in patients most at risk for DCI only.

As expected, milrinone increased cardiac index [9–11]. However, this increase was generally not associated with a higher mean CBF, as opposed to what has been suggested elsewhere [9]. As a consequence, reduction of DCI occurrence with intravenous milrinone administration [5–7, 19] cannot be explained by such systemic effects, *i.e.* a putative global increase in CBF secondary to an increase in cardiac output. This result confirms instead the limited covariation between systemic and cerebral macroscopic hemodynamics [29–31]. However and of interest, mean CBF did increase with CI in patients with DCI. We suggest that this supports altered CBF regulation mechanisms in this specific category of patients, as proposed by others [32, 33]. It is likely that, in this context, CI would be directly transmitted to CBF, independently from vascular transmural pressure or cerebral metabolism controlling CBF in physiological conditions [30].

Nonetheless, milrinone did affect cerebral perfusion, and did so differently in the non-DCI and DCI subpopulations. First, mean CBF decreased with the dose of milrinone in the non-DCI subpopulation. Although the absence of correlation to CI may be owing to lack of statistical power, regulation mechanisms dampening CBF in this context of increased CI are a possibility. Second, CoV and rCoV behaved distinctly in non-DCI and DCI patients. It has been shown that whole-brain spatial heterogeneity of perfusion increases before DCI occurs [19]. In our cohort, milrinone dose was associated with a reduction in the CoV of CBF in patients that would not develop DCI, while there was no association in patients presenting DCI. To make sense of a concomitant decrease in CBF and CoV of CBF with milrinone in non-DCI patients, we propose that CBF rehomogenization, measured as a decreased in CoV, may be more adequate to brain metabolism. In these patients only, preserved neurogliovascular coupling [34] would lead to a decrease in mean CBF through autoregulation [30].

Whole-brain spatial heterogeneity can stem from inter-(global) or intra-arterial (regional) ROIs heterogeneity. Global differences could be explained by macrovasospasm causing macroscopic hemodynamical failure. Regional heterogeneity may arise from mesoscopic and/or microscopic vascular alterations leading to local hemodynamical defects, that would propagate proximally, and/or be exacerbated by more proximal stenosis [35]. Whole-brain CoV cannot differentiate between these two origins. Hence, we measured regional heterogeneity, categorizing data into no-DCI ROI from non-DCI patients, no-DCI ROIs from DCI patients, and DCI ROIs.

Results showed that, first, rCoV behaved differently in no-DCI and DCI ROIs, second, that larger rCoV were associated with DCI, and third, that rCoV decreased with milrinone dose in no-DCI ROIs from patients with DCI while it increased in DCI ROIs. This would confirm that meso/microscopic vascular alterations are indeed involved in DCI [35, 36], and that milrinone may, in some instances, rehomogenize cerebral perfusion. In others, milrinone may on the contrary aggravate brain perfusion. Indeed, rCoV in DCI ROIs increased with milrinone dose, putatively because structural alterations prevented CBF to reach vascular subnetworks. As a consequence of auroregulation loss, milrinone would increase mean CBF, which would be very unevenly distributed, causing the observed raise in rCoV. Of note, no-DCI ROIs from patients with DCI tended to initially behave similarly to DCI ROIs, suggesting again that DCI pathophysiology emerges from global mechanisms unrestricted to specific arterial territories [19]. Of interest, the same patterns were observed when substituting CBF for TMax, showing the robustness of the approach.

Finally, temporal evolution of rCoV of CBF with milrinone dose showed distinct patterns in DCI and non-DCI populations. In the latter, rCoV showed relatively high initial values. Introduction of milrinone intravenous infusion stabilized or even decreased rCoV, indicating perfusion spatial homogenization, and milrinone dose was accordingly secondarily lowered. A radically different pattern was observed in the DCI population both at the whole-brain and at the ROI level. There, rCoV started from higher values, as reported previously [19], and we suggest that this reflects early meso/microvascular alterations. rCoV then showed a drop, again in line with previous report [19], and interpreted as early hyperemia. A third-phase showed a gradual increase. In these ROIs, milrinone intravenous infusion did not allow to curb rCoV escalation, and infarction finally constituted. Overall, these results strengthen the observation that perfusion’s spatiotemporal dynamics differ, from first measurements, in non-DCI and DCI patients [18, 19].

This retrospective study is based on prospectively collected data, mitigating classification bias. It is, however, limited by the relatively low number of included patients, such that a classifier could not be built from early CTP parameters dynamics. Nonetheless, we included data from all patients, none were excluded, hence results should be generalizable. As such, whole-brain dynamics are consistent with the observations reported in [19] from a distinct and larger population of patients.

Another concern may arise from the absence of additional parameter incorporation in this study, that could hide confounding factors. However, limiting this exploration to a priori defined parameters, emerging from explicit hypotheses [19, 35–37], strengthens the theoretical results and limits the risk of finding nonexplicative covariables.

Finally and besides the scope of this report, using CTP parameters’ CoV or rCoV for monitoring cerebral perfusion may be of concern, since CTP notably comes with irradiation-related risks. As discussed elsewhere, cost-efficiency modelling on the contrary suggested that early diagnosis of DCI with CTP should improve outcome and improve quality-adjusted life years [19, 38, 39], advocating for its wider use.

Our observations support the major role of perfusion spatial heterogeneity in patients with SAH, strengthening theoretical [35, 37] and experimental [36, 40] work on microcirculation, proposing that ischemia can arise from this heterogeneity, even, or even worse when mean CBF is maintained or increased.

We propose that altered microvascular network leads to local perfusion heterogeneity that can propagate proximally and reflect at the mesoscopic scale, probed with CTP. We suggest a differential response to systemic administration of milrinone, hypothesizing that microscopic behaviors are responsible for the mesoscopic CTP measurements:

- In responder-patients, who will not develop DCI, systemic milrinone tends to homogenize perfusion, as measured with either rCoV of CBF or TMax; this can increase maximal CMRO_2_ [36, 37], and prevent DCI;
- In non-responder-patients, who will develop DCI, systemic milrinone fails at optimizing CBF spatial repartition, and even increases CBF and TMax global and regional spatial heterogeneity, further reducing maximal CMRO_2_ [36, 37].

We hence suggest that rCoV of CBF and TMax can be used, not only as predictors for DCI [18, 19], but also as reporters for response to systemic milrinone treatment. This suggestion may guide a number of future studies. First, we propose that preclinical experiments could explore the microscopical effects of milrinone on vascular, perivascular, glial cells, and platelets [9], and help elucidate the mechanisms for its beneficial effects in patients with SAH [5–8]. Second, theoretical work is needed to formally explain how early microvascular dynamics alterations, which are pathophysiologically relevant [35–37, 40], may reflect at the macroscopic scale, which is clinically accessible [19]. Third, we advocate for the development of nonirradiating, bedside measurement of CBF dynamics, as has recently been demonstrated using contrast-enhanced ultrasonography [41]. Finally, we suggest conducting large-scale clinical studies testing spatial heterogeneity of brain perfusion, using either CTP or non-CTP parameters’ coefficient of variation, as a trigger for therapeutic interventions and as a readout for treatment efficacy, from the initial stage for prophylaxy in high-risk patients, and during the whole course of disease evolution.

## Conclusions

Intravenous infusion of milrinone does not seem to improve cerebral perfusion through systemic effects. It does differently affect patients with SAH depending on their potential clinical evolution. In non-DCI patients, milrinone seems to homogenize CBF and prevent evolution towards DCI. In patients with DCI, it fails to do so, and CBF spatial heterogeneity pathologically increases until infarction. We suggest that future studies may consider spatiotemporal evolution of CBF heterogeneity as both a biomarker and a pathophysiological feature of DCI.

## Supplemental material

Supplemental Figure 1 presents CI and CBF plotted against milrinone dose for three categories of iSVR. Supplemental Figures 2 and 3 present, respectively, rCoV of CBF and Tmax in single ROIs, and CoV of Tmax in whole-brains. Supplemental Table 1 present haemodynamical data for every patients, together with milrinone and norepinephrine doses, and CTP dates.

## Supplementary Information

Below is the link to the electronic supplementary material.

Supplementary file1 (PDF 14551 KB)

## Data Availability

Data are available upon reasonable request addressed to the corresponding author.
