# Supplementary material for: The Effects of Milrinone Systemic Administration on Cerebral Perfusion as Measured with Computed Tomography in Aneurysmal Subarachnoid Hemorrhage Patients
Source: Neurocrit Care. 2026 May 28;45(2):823–32. doi: 10.1007/s12028-026-02559-3 (PMC13642452; doi:10.1007/s12028-026-02559-3)
Supplement: Supplementary file 1 [file 12028_2026_2559_MOESM1_ESM.pdf]

The effects of milrinone systemic administration on cerebral perfusion as measured with computed tomography in aneurysmal subarachnoid hemorrhage patients.

Szabo et al.

Supplemental material

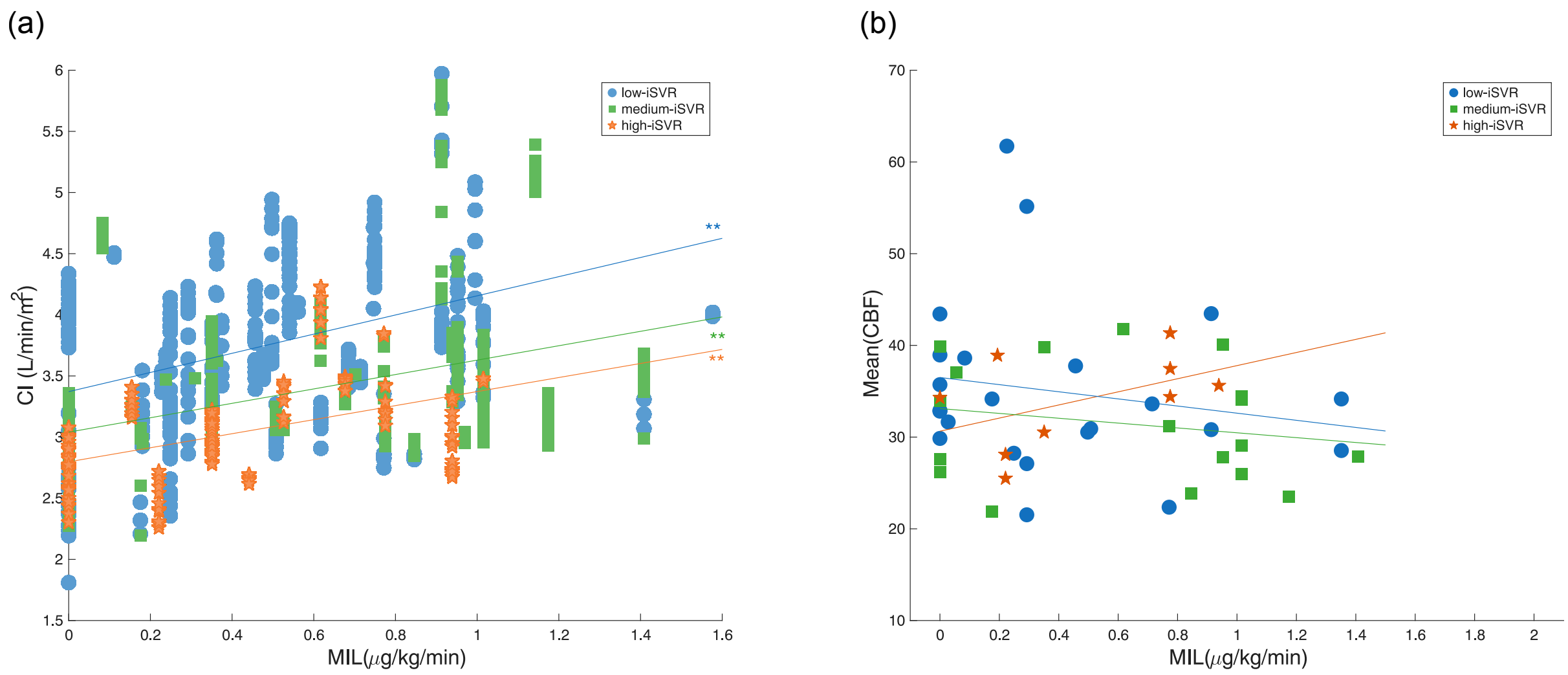

Fig.S1: (a) Relationship between cardiac index (CI) and the dose of milrinone for three baseline levels of iSVR. Blue dots: iSVR<2000; green squares: 2000<iSVR<2500; orange stars: iSVR>2500 dyn.s.cm-5.m^2. Lines indicate linear trends for each dataset. Stars at the end of the line indicate a statistically significant relationship. All three relationships were statistically significant ($p<0.001$) with r^2 values of 0.14, 0.17 and 0.19 respectively. (b) Relationship between mean CBF and the dose of milrinone for three baseline levels of iSVR. Blue dots: iSVR<2000; green squares: 2000<iSVR<2500; orange stars: iSVR>2500 dyn.s.cm-5.m^2. Lines indicate linear trends for each dataset. None of these were statistically significant.

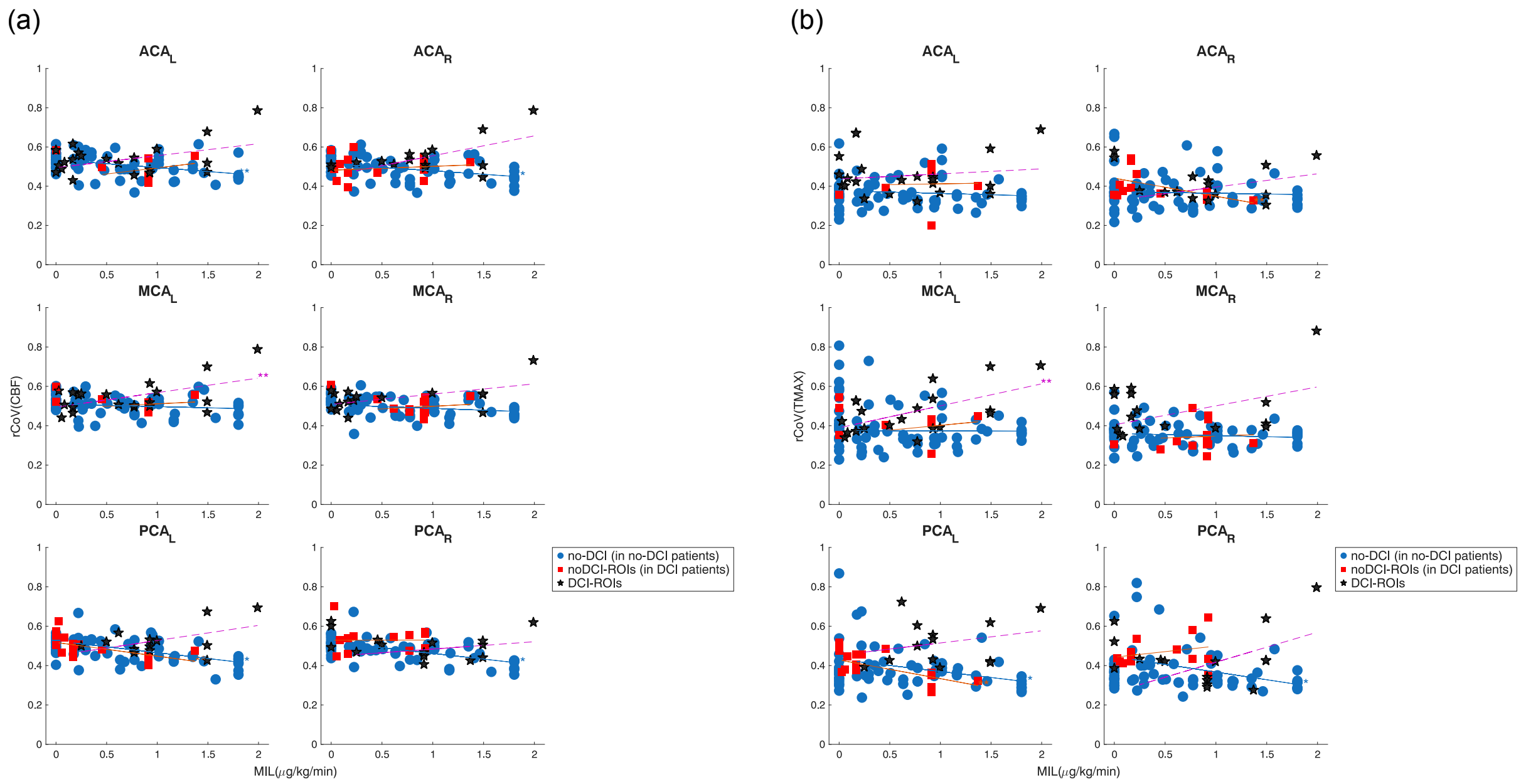

Fig.S2: (a) Local coefficient of variation of CBF, rCoV, as a function of the dose of milrinone per arterial ROI. (b) Local coefficient of variation of TMax, rCoV, as a function of the dose of milrinone per arterial ROI. Blue dots represent data from non-DCI patients, red squares represent no-DCI ROIs from the DCI patients population and black stars represent DCI-ROIs. Lines represent the linear correlation between datasets for the three subpopulations. Stars at the end of the line indicate a statistically significant relationship.

(a) (b)

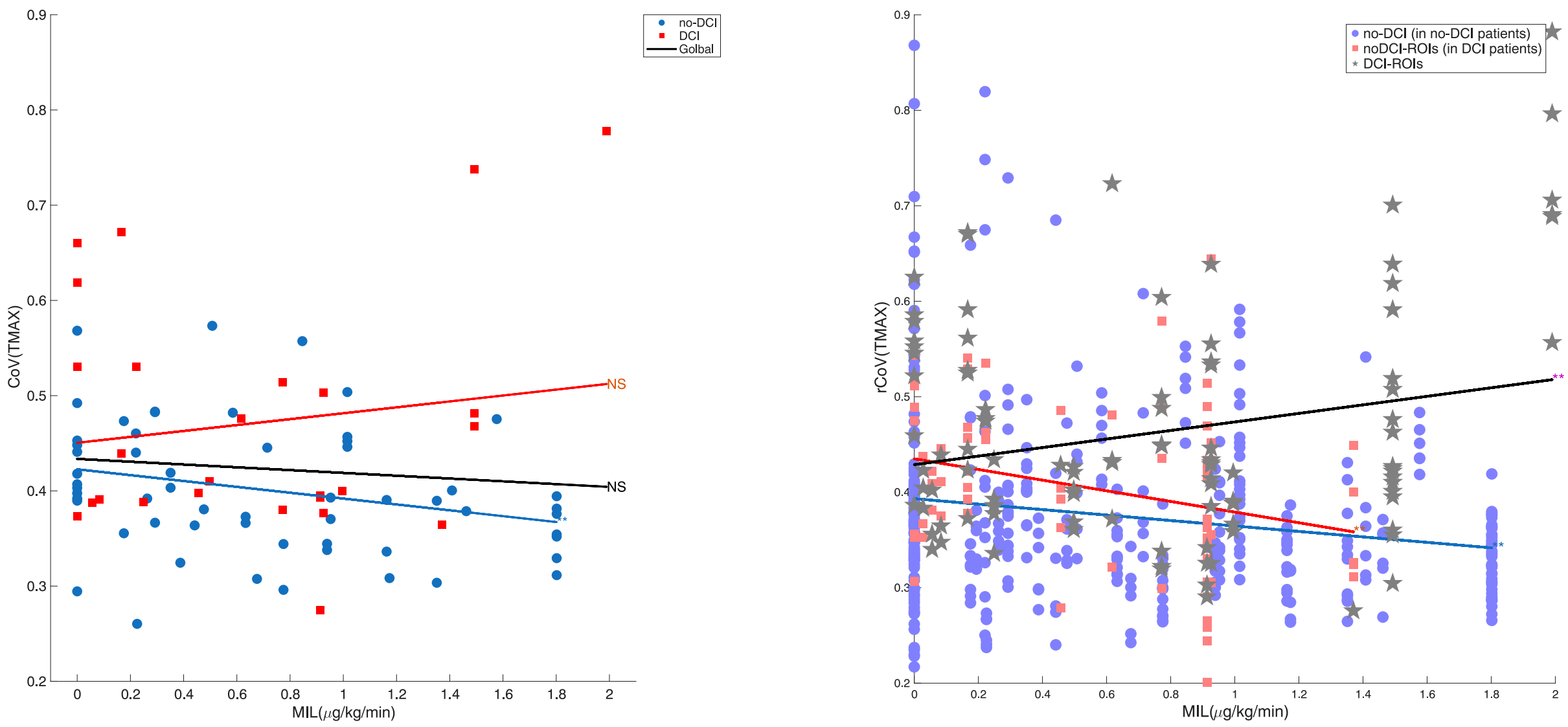

Fig.S3: (a) Whole-brain coefficient of variation of TMax, CoV, as a function of the dose of milrinone. Red squares and blue dots represent data from the DCI and non-DCI subpopulation respectively. (b) Local coefficient of variation of TMax, rCoV, as a function of the dose of milrinone for pooled arterial ROI per categorie. Blue dots represent data from non-DCI patients, red squares represent no-DCI ROIs from the DCI patients population and black stars represent DCI-ROIs. Lines represent the linear correlation between datasets for the three subpopulations. Stars at the end of the line indicate a statistically significant relationship.

Table S1: Summary table of haemodynamical data and drug doses for all patients. In this concise dataset, the median values of haemodynamical metrics are reported per 24 hours slots following SAH symptoms onset. (ICP: intracranial pressure (mmHg); iSVR: systemic vascular resistance (dyn.s.cm-5.m^2); CI: cardiax index (L/min/m^2) ; MAP: mean arterial pressure (mmHg); Noradrenaline and Milrinone doses in ug/kg/min).

**Patient number**	**Day from SAH**	**CI (median)**	**iSVR (median)**	**ICP (median)**	**MAP (median)**	**Noradrenaline dose (median)**	**Milrinine dose (mean)**
P001	1	NA	1299.52	NA	NA	NA	0
P001	3	NA	1915.08	NA	NA	0	0.23
P001	4	NA	1881.92	3.87	92.79	0	1.35
P001	5	NA	1646	5.51	86.63	0.13	1.35
P001	6	4.83	NA	7.8	93.98	0	1.58
P001	7	5.83	NA	7.1	105.67	0.11	1.8
P001	8	4.87	NA	7.01	122.23	0.01	1.8
P001	9	4.68	NA	7.09	111.26	0.07	1.8
P001	10	4.55	NA	7.14	96.28	0.07	1.8
P001	11	NA	NA	5.87	101.92	0.07	1.8
P001	12	NA	NA	NA	NA	0.35	1.8
P001	14	NA	NA	NA	NA	0.07	1.8

P001	17	NA	NA	NA	NA	0.07	0.68
P002	2	NA	NA	NA	NA	NA	0
P002	5	NA	2873.09	NA	NA	NA	0.19
P002	7	NA	2728.5	NA	NA	NA	0.78
P002	8	NA	2910.2	4.92	91.92	0.48	0.78
P002	9	3.19	2631.4	4.35	104.75	0.39	0.78
P002	13	2.62	NA	4.6	105.36	0	1.16
P002	14	3.01	NA	5.49	108.92	0	1.16
P002	16	NA	NA	4.38	96.42	0.07	0.39
P003	1	NA	1791.14	5.23	89.81	0.05	0
P003	3	3.79	1413.92	7.43	90.03	0.19	0.46
P003	6	3.92	1881.11	7.59	116.38	0.1	0.91
P003	8	5.6	1931.98	9.25	81.87	0.18	0.91
P003	10	5.4	NA	4.1	112.26	0.01	1.37
P003	11	5.4	NA	5.35	111.43	0	0.91
P003	14	3.99	NA	5.13	107.75	0	0.91

P004	1	NA	1741.11	NA	NA	0.14	0.03
P004	3	NA	2176.15	8.28	82.28	0.06	0.06
P004	4	4.53	1976.82	9.16	100.02	0.01	0.08
P004	6	4.03	NA	12.56	114.13	0.01	0.17
P004	7	3.83	NA	7.6	90.56	0.01	0.22
P004	9	3.57	NA	7.03	86.23	0.01	0.17
P004	10	NA	NA	59.41	123.01	0	0.17
P005	1	3.1	1964.74	10.64	92.77	0	NA
P005	3	4.05	2404.97	9.87	117.52	0	0.62
P005	4	3.83	2495.44	6.54	100.98	0	0.77
P005	5	2.86	1598.43	4.57	101.14	0.04	0.77
P005	7	3.33	NA	8.2	99.73	0	0.93
P005	8	4.16	NA	8.33	86.79	0.08	0.93
P005	11	NA	NA	NA	NA	NA	0.93
P006	3	NA	2954.36	9.8	90.85	0.66	0.22
P006	6	2.61	3030.8	6.36	101.29	0.09	0.22

P006	8	2.69	NA	4.38	84.49	0.09	0.44
P007	0	2.9	1302	11.97	81.57	0.06	0.29
P007	1	3.61	1237.39	13.25	79.55	0.06	0.29
P007	2	3.84	1434.13	8.89	83.9	0.02	0.29
P007	4	4.3	1413.75	20.11	99.33	0	0
P007	5	4	1296.36	11.24	93.15	0	0
P007	7	4.87	NA	10.87	87.27	0.23	0.58
P007	9	5.4	NA	13.9	92.33	0.51	1.46
P007	12	4.99	NA	10.11	87.66	0.07	0
P007	14	NA	NA	7.34	85.95	NA	0
P007	17	NA	NA	NA	NA	NA	0
P007	21	NA	NA	NA	NA	NA	0
P008	2	NA	1703.22	8.68	79.47	0.05	0
P008	3	2.86	1704.82	13.61	83.02	0.13	0.51
P008	4	3	2111.16	6.31	88.67	0.08	0.85
P008	6	3.69	2169.73	5.89	98.56	0.01	1.02
P008	9	3.06	2185.82	10.05	91.57	0.03	1.02

P008	11	3.32	2135.82	7.62	99.48	0.08	1.02
P008	13	3.17	2365.45	7.23	96.81	0.11	1.02
P010	2	NA	2588.07	7.09	83.94	0	0
P010	5	2.74	2822.68	9.41	98.95	0	0.94
P010	7	3.34	2333.18	9.95	109.58	0	1.17
P010	9	3.68	2343.17	12.16	104.2	0	1.41
P010	11	2.96	NA	6.18	113.91	0	0.94
P011	3	3.09	1652.31	8.32	91.4	0.19	0.25
P011	6	3.71	1780.65	6.64	100.5	0.37	0.5
P011	8	3.74	NA	11.73	104.2	0.02	1
P011	10	4.89	NA	25.51	87.19	1	1.49
P011	11	4.68	NA	15.3	80.41	1.02	1.49
P011	12	4.02	NA	7.93	93.93	0.6	1.49
P011	13	4.41	NA	17.25	96.84	0.42	1.99
P012	0	NA	1937.36	6.8	90.45	0.08	0.18
P012	1	2.5	2016.57	7.58	82.13	0.07	0.18
P012	5	3.71	2229.84	8.66	99.24	0	0.35

P012	8	3.12	2851	10.2	84.4	0	0.35
P012	11	3.25	NA	3.38	87.6	0	0.26
P012	18	2.56	NA	6.47	95.28	NA	0
P013	2	3.04	2168.09	8.13	88.67	0.19	0
P013	4	3.18	2148.55	10.86	100.57	0	0
P013	5	3.45	1781.45	7.12	113.42	0.02	0.71
P013	7	3.72	2230.05	9.63	99.9	0	0.95
P013	9	4.35	2025.8	12.37	99.92	0	0.95
P013	12	4.52	NA	7.1	94.92	0	0.48
P013	15	3.9	NA	9.46	110.51	NA	0
P015	0	NA	1856.09	NA	NA	NA	0
P015	3	3.58	NA	13.69	77.97	0.19	0
P015	4	3.3	NA	8.23	77.04	0.32	0
P015	6	NA	NA	19.71	78.94	1.37	0
P017	3	NA	1548.27	8.66	101.18	0	NA
P017	8	2.42	2396.62	21.75	79.32	1.34	0
P017	10	3.31	2349.13	13.36	90.21	0.27	0

P018	3	NA	2070.32	NA	NA	NA	NA
P018	8	3.76	NA	NA	105.81	0	0.63
P018	11	NA	NA	NA	NA	0	0.63
